# Early childhood language outcomes after arterial switch operation: a prospective cohort study

**DOI:** 10.1186/s40064-016-3344-5

**Published:** 2016-09-29

**Authors:** Matt S. Hicks, Reginald S. Sauve, Charlene M. T. Robertson, Ari R. Joffe, Gwen Alton, Dianne Creighton, David B. Ross, Ivan M. Rebeyka, for the Western Canadian Complex Pediatric Therapies Follow-up Group

**Affiliations:** 1 Department of Pediatrics, University of Alberta, Edmonton, AB Canada; 2 Department of Pediatrics, University of Calgary, Calgary, AB Canada; 3 Pediatric Rehabilitation Outcomes Unit, Glenrose Rehabilitation Hospital, Edmonton, AB Canada; 4 Department of Surgery, University of Alberta, Edmonton, AB Canada; 5 Department of Community Health Sciences, Cumming School of Medicine, University of Calgary, Calgary, AB Canada

**Keywords:** Transposition of great vessels, Follow-up studies, Child development, Thoracic surgery, Cohort studies

## Abstract

**Background:**

Developmental and language outcomes at 2 years of age of children who had arterial switch operation (ASO) for transposition of the great arteries 2004–2010 are described.

**Methods:**

In this prospective cohort study, 91/98 (93 %) children who underwent ASO were assessed at 2 years of age with the Bayley Scales of Infant & Toddler Development—3rd Edition. Outcomes were compared by patient and perioperative variables using bivariate and multivariate regression analyses to identify predictors of language delay.

**Results:**

Infants without ventricular septal defect (VSD) (n = 60) were more likely to be outborn (73 vs 58 %, p = 0.038), require septostomy (80 vs 58 %, p = 0.026), have a shorter cross clamp time (min) (62.7 vs 73.0, p = 0.019), and a lower day 1 post-operative plasma lactate (mmol/L) (3.9 vs 4.8, p = 0.010). There were no differences in cognitive, motor and language outcomes based on presence of a VSD. Language delay (<85) of 29 % was 1.8 times higher than the normative sample; risk factors for this in multivariate analyses included <12 years of maternal education (AOR 19.3, 95 % CI 2.5–148.0) and cross-clamp time ≥70 min (AOR 14.5, 95 % CI 3.1–68.5). Maternal education <12 years was associated with lower Language Composite Scores (−20.2, 95 % CI −32.3 to −9.1).

**Conclusions:**

Outcomes at 2 years of age in children who undergo ASO are comparable to the normative sample with the exception of language. There is a risk of language delay for which maternal education and cross-clamp duration are predictors. These findings suggest that focused post-operative early language interventions could be considered.

## Background

Arterial switch operation (ASO) is the main surgical modality for uncomplicated transposition of the great arteries (TGA) (Freed et al. 2006). Developmental and language delays have been described in previous cohorts with TGA following ASO, however there has since been evolution of perioperative management strategies that may have led to further improvement in outcomes (Bellinger et al. 1999; Hövels-Gürich et al. 2002; Williams 2003; Freed et al. 2006). Overall long term neurocognitive, health and motor outcomes have improved over time and in most recent studies these are comparable to the general population with the exception of differences in executive function, academic achievement, behaviour and motor skills (Bellinger et al. 1995, 1999; Gaynor et al. 2007). Language delay out of keeping with overall neurocognitive function has been reported in several cohorts following repair of TGA over the last 3 decades (Bellinger et al. 1999; Hövels-Gürich et al. 2002; Bellinger et al. 2003; Neufeld et al. 2008). A report of a cohort from a previous surgical era at our center found increased rates of intellectual disability, autism spectrum disorder and language delay at 5 years of age (Neufeld et al. 2008). Social class, cardio-pulmonary bypass (CPB) duration, clinical and subclinical seizures, and ventricular septal defect (VSD) have been identified as predictors of developmental outcome (Bellinger et al. 1995; Rappaport et al. 1998; Hövels-Gürich et al. 2002; Wypij et al. 2003; Beca et al. 2009). In this article the early neurocognitive and language outcomes for children undergoing ASO in a tertiary center in the period 2004–2010 are described and children were stratified by presence or absence of a VSD to determine if this remains a risk factor for poorer outcome.

## Methods

This cohort study enrolled 102 consecutive children who underwent ASO for repair of TGA at Stollery Children’s Hospital, Edmonton, Alberta in the period July 2004 to June 2010. This study included all children with an anatomic diagnosis of TGA with or without VSD. Cardiac surgery was performed by two cardiac surgeons (DBR, IMR). Operative procedures included full-flow cardiopulmonary bypass (CPB), moderate hypothermia and deep hypothermic circulatory arrest (DHCA) in 66 % of cases and a minimum hematocrit value of 0.25 %. Use of DHCA was generally reserved for the short period required to close the ASD. Venous cannulation strategy was based upon surgeon preference. Intraoperative lactate measurements were not routinely collected. Perioperative electroencephalograms (EEGs) were not obtained unless there was clinical concern of seizure. Near-infrared spectroscopy (NIRS) has recently been introduced to the clinical environment to measure cerebral oxygenation but these data are not available for the entire cohort. Perioperative and demographic characteristics were collected prospectively and included the following: demographics (sex, race, socioeconomic status—Blishen, maternal education, delivery at a hospital outside of the region of the operating site or outborn, and year of operation), birth characteristics (antenatal diagnosis, gestational age, birth weight, use of balloon atrial septostomy (BAS), and age at operation), peri-operative measures of illness severity (modified inotrope score, highest plasma lactate value, total days of ventilation, presence of convulsions, sepsis, renal insufficiency as defined by need for dialysis or creatinine >100 μmol/L and total hospital days at the operative site), and operative characteristics (CPB time, cross-clamp time, and use of DHCA) (Blishen et al. 1987). The postoperative modified inotrope score was calculated as per Wernovsky and colleagues (Wernovsky et al. 1995; Mackie et al. 2013). All children underwent genetic testing including testing for 22q11 deletion.

## Outcome assessment

As previously described, children in the cohort were assessed at 6–10 months and at 18–24 months of age by multidisciplinary teams at one of 5 centers participating in the Western Canadian Complex Pediatric Therapies Follow-up Group: Calgary and Edmonton, Alberta; Regina and Saskatoon, Saskatchewan; and Winnipeg, Manitoba (Robertson et al. 2011). At the 18–24 month follow up visit children were assessed using several psychological tools, had a clinical interview and physical examination by a pediatrician experienced in developmental follow up, and completed standardized parental-report questionnaires (history of hospitalization, family socioeconomic status) as previously described (Neufeld et al. 2008). Hearing was evaluated by certified audiologists. The Bayley Scales of Infant & Toddler Development—3rd Edition (Bayley-III), a measure of early child development, was administered by experienced pediatric psychologists or psychometrists under the supervision of a psychologist (Bayley 2006). The Bayley-III yields Cognitive, Motor and Language Composite Scores each expressed as a standard score with a mean of 100 and standard deviation (SD) of 15. Subscales include Receptive and Expressive Communication and Fine and Gross Motor with a mean of 10 and a SD of 3.

### Statistical analysis

All statistical analyses were performed with Intercooled STATA Version 12.0 (College Station, Texas) (2011). All tests were two-sided (where applicable) and significance was defined as *p* value <0.05. A correction for multiple comparisons was not applied given the exploratory nature of this work. Bivariate analyses, χ^2^ analyses or Fisher’s exact test where appropriate, were used to compare Cognitive, Motor and Language Scores of the Bayley-III by presence or absence of VSD and perinatal and maternal characteristics. Continuous variables were compared between groups using Student’s t test (two-sided). Descriptive statistics and bivariate analysis were used to identify differences based on the presence or absence of a VSD. Cognitive, Motor and Language Composite Scores were compared to those of the Bayley-III normative sample where 2.27 % of the sample had a score <70 and <85 (15.86 %) (Bayley 2006).

Multivariate linear and logistic regression models were developed with a dependent variable of the Language Composite of the Bayley-III. In addition, models were also developed with dependent variables of Receptive Communication or Expressive Communication scores less than 7. Independent variables were included in the models if they were significantly associated with the outcome at p < 0.2 in preliminary univariate analysis and then removed in a backwards stepwise fashion to yield a parsimonious model. Multicolinearity was screened for prior to inclusion in multivariate models. Adjusted odds ratios (for language delay), effect sizes (for Language Composite Score) and 95 % confidence intervals are reported.

Research ethics board approval was obtained from each site, and all parents or guardians provided written informed consent. Funding agencies had no role in data interpretation.

## Results

### Description of cohort

**Fig. 1:**
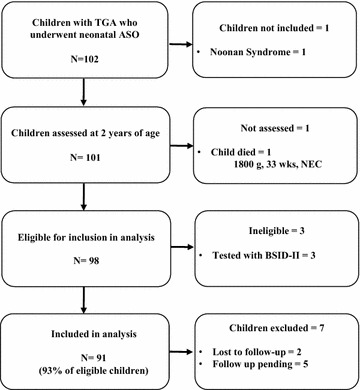
Study flowchart of recruitment of participants between July 2004 and June 2010 for neonates who underwent neonatal arterial switch operation (ASO) for transposition of the great arteries (TGA)

A flowchart of recruitment participation and loss to follow up is presented in Fig. 1. A total of 103 children underwent ASO during the time period. One child was excluded from the cohort as the child had an identified genetic syndrome and one child who underwent ASO died at day 106 of life of complications of preterm birth. Three children were not eligible for inclusion in this analysis as they were assessed with the Bayley Scales of Infant Development—2nd Edition. Of those eligible for inclusion, 2 children were lost to follow-up and 5 children still had results pending at time of the analysis. In this study 91 of 98 eligible children (93 %) had follow up outcomes with Bayley-III and were included in this analysis. There were no differences in demographic variables between those who were included in the analysis and those who were excluded.

### Perioperative and demographic characteristics

Demographic and perioperative characteristics of neonates are presented in Tables 1 and 2. The majority of children did not have a VSD (n = 60). Of those a lower proportion were outborn (52 vs. 73 %, p = 0.038) and had a pre-operative BAS (58 vs. 80 %, p = 0.026). Children with VSD had a longer cross clamp time (73.9 vs. 62.7 min, p = 0.019), a higher post-operative lactate on day 1 (4.8 vs 3.9 mmol/L, p = 0.010) and were more likely to require dialysis or have a creatinine >100 μmol/L (23 vs. 7 %, p = 0.041). No children in this cohort received extracorporeal membrane oxygenation, cardiopulmonary resuscitation, ventricular-assist device or heart transplant.

**Table 1:** Preoperative and demographic characteristics of neonates requiring arterial switch operation for transposition of the great arteries in the period 2004–2010

Variables	Total N = 91 n (%)	Ventricular septal defect		p value
		No VSD N = 60 n (%)	VSD N = 31 n (%)	
*Demographic and birth characteristics*				
Male	61 (67 %)	38 (63 %)	23 (74 %)	0.30
Antenatal diagnosis: yes	27 (30 %)	17 (28 %)	10 (32 %)	0.70
Birth gestation (weeks)	39.0 (1.8)	39.1 (1.9)	38.8 (1.5)	0.60
Birth weight (g)	3367.6 (569)	3370.1 (597)	3363.0 (517)	0.96
Apgar at 5 min	8.1 (1.2)	7.9 (1.3)	8.3 (0.9)	0.24
Socioeconomic index (Blishen)	46.3 (13.9)	45.6 (12.9)	47.7 (15.8)	0.50
Total years of mother’s schooling	14.1 (3.3)	13.6 (2.9)	15.1 (3.9)	0.06
Guardianship				
Parents	81 (89 %)	52 (87 %)	29 (94 %)	
Mother	6 (7 %)	4 (7 %)	2 (6 %)	
Father	1 (1 %)	1 (2 %)	0 (0 %)	
Other	3 (3 %)	3 (5.0 %)	0 (0 %)	1.00
Race				
European/white	70 (84 %)	45 (80 %)	25 (93 %)	
Original peoples	2 (2 %)	2 (4 %)	0 (0 %)	
Other	11 (13 %)	9 (16 %)	2 (7 %)	0.32
Delivery at hospital outside of the region of the operating site	60 (66 %)	44 (73 %)	16 (52 %)	0.04
*Preoperative characteristics*				
Pre-operative septostomy	66 (73 %)	48 (80 %)	18 (58 %)	0.03
Modified inotrope score	7.7 (9.7)	7.5 (9.8)	8.1 (9.6)	0.78
Plasma lactate (mmol/L)	3.4 (3.1)	3.3 (3.3)	3.6 (2.7)	0.65
Days ventilated (days)	5.3 (4.7)	5.2 (3.1)	5.5 (6.9)	0.85
Highest serum creatinine (μmol/L)	62.6 (19.1)	62.2 (17.1)	63.3 (22.8)	0.80
Sepsis (blood culture confirmed)	3 (3 %)	3 (5 %)	0	0.55
Hydrocortisone cumulative exposure (mg/kg)	4.2 (12.8)	4.8 (14.8)	3.1 (7.7)	0.49

**Table 2:** Perioperative characteristics of neonates who required arterial switch operation for transposition of the great arteries in the period 2004 to 2010

Variables	Total N = 91 n (%)	Ventricular septal defect		p value
		No VSD N = 60 n (%)	VSD N = 31 n (%)	
Operative				
Year at surgery	2007.3 (2.2)	2007.3 (1.9)	2007.4 (2.6)	0.80
Age at surgery (days)	11.5 (14.8)	9.1 (3.8)	16.2 (24.4)	0.12
Weight at surgery (kg)	3.485 (0.669)	3.445 (0.607)	3.562 (0.781)	0.43
Cardiopulmonary bypass time (min)	120.6 (39.8)	116.8 (32.9)	128.0 (50.3)	0.27
Cross-clamp time (min)	66.5 (18.5)	62.7 (14.0)	73.9 (23.6)	0.02
Use of deep hypothermic circulatory arrest	60 (66 %)	42 (70 %)	18 (58 %)	0.26
Need for re-cardiopulmonary bypass during first OR	7 (8 %)	3 (5 %)	4 (13 %)	0.22
Post-operative, day 1				
Modified inotrope score	8.7 (7.2)	8.5 (7.5)	9.1 (6.8)	0.70
Plasma lactate (mmol/L)	4.2 (1.6)	3.9 (1.6)	4.8 (1.5)	0.01
Time for lactate to return to 2 or less (h)	10.9 (8.4)	10.3 (8.9)	12.0 (7.3)	0.36
Highest serum creatinine (μmol/L)	51.5 (12.3)	50.3 (11.9)	53.8 (13.0)	0.19
Post-operative, day 2–5				
Modified inotrope score	7.7 (7.7)	7.1 (6.7)	8.7 (9.3)	0.41
Plasma lactate (mmol/L)	2.2 (1.1)	2.2 (1.2)	2.3 (0.8)	0.82
Serum creatinine (μmol/L)	61.5 (22.2)	61.5 (24.3)	61.3 (17.9)	0.97
Post-operative, day 6+				
Modified inotrope score	1.2 (2.6)	1.2 (2.6)	1.4 (2.6)	0.74
Plasma lactate (mmol/L)	1.1 (0.4)	1.1 (0.35)	1.2 (0.49)	0.21
Serum creatinine (μmol/L)	47.0 (20.7)	45.2 (20.9)	50.4 (20.3)	0.26
All post-operative time				
Ventilation (days)	6.2 (3.5)	6.0 (3.5)	6.7 (3.4)	0.38
Admission to PICU (days)	10.8 (5.0)	10.5 (4.9)	11.4 (5.2)	0.37
Overall/any time				
All hospital days (SCH)	19.1 (8.4)	18.1 (8.3)	21.0 (8.4)	0.12
Convulsions	6 (7 %)	2 (4 %)	3 (14 %)	0.41
Ventilation (days)	11.0 (5.5)	11.1 (5.4)	10.7 (5.7)	0.73
Sepsis pre or post-operative (blood culture positive)	7 (8 %)	3 (6 %)	3 (10 %)	0.42
Dialysis	4 (4 %)	1 (2 %)	3 (10 %)	0.11
Dialysis or creatinine >100 (μmol/L)	11 (12 %)	4 (7 %)	7 (23 %)	0.04

Brain imaging, either ultrasound or magnetic resonance imaging, were not adequate for inclusion in this analysis as these were not consistently obtained perioperatively. In addition, information on seizures could not be rigorously considered here because electroencephalograms (EEGs) were not consistently obtained.

#### Neurocognitive outcomes

**Fig. 2:**
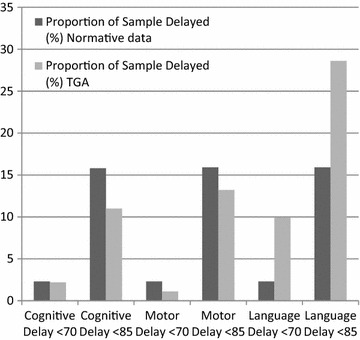
Developmental outcomes at 2 years of age on the Bayley Scales of Infant & Toddler Development—3rd Edition (Bayley-III) after neonatal arterial switch operation for transposition of the great arteries compared to Bayley-III normative data

There were no differences in cognitive, motor and language outcomes based on presence of VSD (Tables 3, 4). Only 3 % of children had a Cognitive score <70 (2SD below the mean) while 11 % had a Language score <70 and 4 % had a Motor score <70. 12 % of children had Cognitive and Motor scores <85 (1SD below the mean). Compared to the normative sample for the Bayley-III, children with TGA were 4.7 times more likely to have a Language score <70 and 1.8 times more likely to have a Language score <85 (Fig. 2).

**Table 3:** Mean scores on the Bayley Scales of Infant & Toddler Development—3rd Edition after arterial switch operation for transposition of the great arteries for children with and without ventricular septal defect (n = 91)

Variable	Total n = 91 mean (SD)	Ventricular septal defect		p value
		No VSD n = 60 mean (SD)	VSD n = 31 mean (SD)	
Bayley-III				
Cognitive composite	96.9 (12.7)	98.4 (13.1)	93.8 (10.5)	0.10
Language composite	92.6 (17.0)	92.3 (18.6)	93.2 (13.)	0.81
Motor composite	99.0 (12.8)	98.9 (13.7)	99.3 (11.2)	0.89
Subscales				
Receptive communication	9.1 (3.0)	9.2 (3.3)	8.8 (2.4)	0.52
Expressive communication	8.4 (3.2)	8.2 (3.3)	8.8 (2.8)	0.40
Fine motor	10.8 (2.7)	10.7 (2.7)	11.1 (2.7)	0.57
Gross motor	9.0 (2.1)	9.0 (2.3)	8.9 (1.9)	0.81

**Table 4:** Development outcomes at 2 years of age as determined by the Bayley Scales of Infant & Toddler Development—3rd Edition after arterial switch operation for transposition of the great arteries (n = 91)

Outcome	Total n = 91	VSD		Fisher’s exact, p
		No VSD n = 60	VSD n = 31	
Cognitive				
<70	2 (2.2 %)	1 (1.7 %)	1 (3.2 %)	1.00
<85	10 (11.0 %)	6 (10.0 %)	4 (12.9) %	0.73
Language				
<70	9 (9.9 %)	8 (13.3 %)	1 (3.2 %)	0.16
<85	26 (28.6 %)	19 (31.7 %)	7 (22.6 %)	0.46
Motor				
<70	1 (1.1 %)	1 (1.7 %)	0 (0 %)	1.00
<85	12 (13.2 %)	9 (15.0 %)	3 (9.7 %)	0.74
Cognitive <7	9 (9.9 %)	6 (10.0 %)	3 (9.7 %)	1.00
Receptive communication <7	22 (24.2 %)	17 (28.3 %)	5 (16.1 %)	0.30
Expressive communication <7	25 (27.5 %)	18 (30.0 %)	7 (22.6 %)	0.62
Fine motor <7	4 (4.4 %)	4 (6.7 %)	0 (0 %)	0.30
Gross motor <7	7 (7.7 %)	5 (8.3 %)	2 (6.5 %)	1.00

#### Language outcomes

A parsimonious logistic regression model was developed with an outcome of Language Composite <85) on the Bayley-III and adjusted odds ratios (AOR) for patient and perioperative characteristics for children are presented in Table 5. The final model has an overall pseudo R^2^ of 0.32. Maternal education less than 12 years (AOR 19.3, 95 % CI 2.5–148.0), cross-clamp time (AOR 14.5, 95 % CI 3.1–68.5), DHCA (AOR 5.8, 95 % CI 1.3–26.7), and days of ventilation (AOR 1.2, 95 % CI 1.0–1.3) were risk factors for language delay. Female sex, ventricular septal defect and birth at the surgical center were controlled for in the model although not significant predictors of delay. Separate multiple logistic regression models were also developed with receptive or expressive Language scores less than 7 as the outcome of interest. Both models had maternal education less than 12 years as the main predictor of delay (data not shown).

**Table 5:** Adjusted odds ratios (AOR) for patient and perioperative characteristics for children with language composite <85 on the Bayley Scales of Infant & Toddler Development—3rd Edition at 2 years of age following arterial switch operation for transposition of the great arteries

Characteristic	Delayed (n = 26) n (%)	Within normal limits (n = 65) n (%)	AOR (95 % CI)
Patient characteristics			
Female sex	10 (38.5)	20 (30.8)	2.7 (0.7–9.7)
Maternal education: less than 12 years	7 (26.9)	2 (3.1)	19.3 (2.5–148.0)
Ventricular septal defect present	7 (26.9)	24 (36.9)	0.4 (0.1–1.5)
Born at surgical center	4 (15.4)	27 (41.5)	0.3 (0.1–1.3)
Perioperative characteristics			
Cross-clamp time ≥70 min	13 (50.0)	20 (30.8)	14.5 (3.1–68.5)
DHCA	19 (73.1)	41 (63.1)	5.8 (1.3–26.7)
Days of ventilation (for each additional day of ventilation)	13.6 (6.7)	10.0 (4.5)	1.2 (1.0–1.3)

A parsimonious linear regression model was generated with an outcome of language delay (Language Composite Score <85). The model included maternal education and cross clamp time and had an overall R^2^ of 0.203. The language constant was 97.6 (95 % CI 93.2–101.9) and children who had mothers with less than a Grade 12 education had Language Composite Scores that were 20.2 points lower (95 % CI −32.4 to −9.1). Children with a cross clamp time of 70 min or greater had a Language Composite Score 8.0 points lower (95 % CI −15.0 to −1.1). Separate multiple linear regression models were also developed with receptive or expressive language as the continuous outcome of interest. Both of the parsimonious models constructed had maternal education less than 12 years as the main predictor of delay (data not shown).

## Discussion

As previously described in recent cohorts, children with TGA who undergo ASO have a low mortality rate as was found in this cohort (Bellinger et al. 1995, 1999; Losay et al. 2001; Bellinger et al. 2003; Neufeld et al. 2008). In addition, as previously noted the children at our center have good preschool neurocognitive, motor and overall health outcomes which are in keeping with the published literature (Bellinger et al. 1995, 1999; Dunbar-Masterson et al. 2001; Hövels-Gürich et al. 2002; Bellinger et al. 2003; Karl et al. 2004; Freed et al. 2006; Creighton et al. 2007; Neufeld et al. 2008). Only 2 % of children had Cognitive scores <70 and only 1 child had a Motor score <70, scores below 70 would be concerning for the later development of cognitive or motor disabilities. Consistent with cohorts at several centers and over several surgical eras approximately 25 % of children in this study had language delay without evidence of co-occurring delay in cognitive abilities (Bellinger et al. 1995, 1999; Hövels-Gürich et al. 2002; Karl et al. 2004; Neufeld et al. 2008). The children in this cohort were delayed in comparison to the normative sample from the BSID-III in expressive and receptive communication. Of note, the BSID-III normative sample included children who per born preterm and children with disabilities. In a previous cohort at this center for children undergoing surgery for TGA 1996–2004, 26 % had speech or language difficulties at 5 years of age (Neufeld et al. 2008). Children with early language delay are at risk in adolescence and adulthood of poor developmental and mental health outcomes including communication, cognitive, behavioral, social, academic and mental health difficulties (Bashir and Scavuzzo 1992; Johnson et al. 1999; Beitchman et al. 2001; Young et al. 2002; Durkin and Conti-Ramsden 2007). Multiple regression analysis identified maternal education, cross clamp time, requirement for DHCA and total days of ventilation as predictors of language delay. However, only a third of the variance in Language scores was explained by the variables in our model. Multiple regression models with receptive or expressive Language delay as the dependent variable yielded similar parsimonious models. A previous study at our center found maternal education was an important predictor of neurocognitive outcome but variables in regression models only explained roughly 30 % of the variance in outcome (Neufeld et al. 2008). Only 9 mothers in the current study had education less than 12 years so this appears to represent a small subset with an important risk factor for language delay that does not explain the delays seen in the majority of patients. The consistency of language delay across surgical eras and the relatively small amount of variance explained by perioperative variables suggests a considerable influence of currently unidentified risk factors on neurodevelopmental outcome. These risk factors may include the impact of the quality of caregiver-child interaction and parental perception of chronic illness on developmental trajectory.

Deficits often do not become apparent or identifiable until adolescence when demands in social interaction, communication, cognition and executive function increase (Bellinger et al. 1995, 2003; Ovadia et al. 2000; Bellinger and Newburger 2014). Ongoing assessment of neurocognitive outcomes for this cohort is important as a previous surgical era at our center found a higher than expected rate of Autism Spectrum Disorder (ASD) (Neufeld et al. 2008). Approximately 11 % of children had an Intellectual Disability (ID) and 5.5 % had an Autism Spectrum Disorder (Neufeld et al. 2008). Previous studies have also found increased rates of deficits in Executive Function and in symptoms of Attention-Deficit Hyperactivity Disorder (ADHD) (Bellinger et al. 2003). The children in the current cohort were too young for definitive assessment for ID, ASD, ADHD and specific deficits in executive function and this will need to be assessed in the future. A 2012 scientific statement from the American Heart Association which was approved by the American Academy of Pediatrics concluded that “Periodic developmental surveillance, screening, evaluation and re-evaluation throughout childhood may enhance identification of significant deficits, allowing for appropriate therapies and education to enhance later academic, behavioral, psychosocial, and adaptive functioning” (Marino et al. 2012). The results of previous cohorts as well as this cohort highlight the need to continue to assess developmental outcomes for children with TGA (Bellinger et al. 2003; Neufeld et al. 2008; Beca et al. 2009).

A recent meta-analysis found that early interventions in the preterm child can lead to improvements in early and mid-term cognitive outcomes however these benefits were not sustained at school age (Spittle et al. 2012). Children with phonological and expressive vocabulary difficulties may benefit from speech and language intervention, although the most effective model of delivery is not clear (Law et al. 2003; Cirrin et al. 2010; Spittle et al. 2012; Moharir et al. 2014). Children may benefit from focused post-operative early intervention to improve language and developmental outcomes. In particular, children with mothers with education less than 12 years could be considered for targeted intervention.

As previously reported, cross-clamp time is also a predictor of neurocognitive outcome, underscoring the importance of minimizing interruptions in circulation. The mean cross-clamp times compare favorably with those reported in other studies (Bellinger et al. 1995). Cross-clamp time, DHCA and days of ventilation are most likely markers of complexity of surgical intervention and severity of illness. Presence of a VSD was not associated with poorer outcome in this study.

Balloon atrial septostomy was also not a predictor of outcome in this study but was in previous cohorts at our center that considered 5 year outcomes (Neufeld et al. 2008). Neonatal hypoxia and abnormal fetal circulation contribute to brain insult (du Plessis and Volpe 2002). In a recent study the rates of peri-operative brain injury as determined by magnetic resonance imaging (MRI) were similar between infants with TGA, hypoplastic left heart syndrome and pulmonary atresia with no difference in rate of injury by need for BAS (Beca et al. 2009). Overall rates of brain injury appeared to be higher in infants with intact VSD although this did not reach statistical significance (Beca et al. 2009).

The mean age at surgery in this group was 9.1 days in neonates without a VSD and 16.2 for those with a VSD. A recent review of 140 infants with d-TGA who had ASO between 2003 and 2012 at a major surgical center in New York found a significant increase in major morbidity and healthcare costs for every day of delay in surgery beyond day 3 of life (Anderson et al. 2014; Karamlou 2014). The authors concluded that “ASO ought to be performed in preterm and term infants no later than the first week of life”. They identified day 3 as the optimal day for surgery with increased risk of major morbidity if surgery was performed day 1–3 with larger increased risk from day 4 and beyond. In addition, earlier operation could reduce the rate of BAS which was high in this group at 73 %. A recent meta-analysis found a BAS rate in TGA of between 20 and 54 % (Doshia et al. 3023). The authors concluded that BAS remains an important option for patients with restrictive interatrial communication who will have a delay in surgery due to transport or lack of access to a surgical team (Doshia et al. 2012). Time to surgery was not a risk factor for language delay in this cohort. Additional prospective studies are required to understand the optimal timing for surgery.

The relatively consistent rates of brain injury regardless of specific type of congenital heart disease in that study would be consistent with a brain injury acquired secondary to hypoxia. Brain imaging, either ultrasound or magnetic resonance imaging, were not included in this analysis as this is not consistently obtained perioperatively. Pre and post-operative MRIs to identify timing of acquisition of white matter injury and potential identification of children at risk for delay could be considered.

Information on clinical and nonclinical seizures could not be rigorously analyzed as EEGs were not consistently obtained. Previous studies found that electrographic seizures are common after heart surgery and children with clinical seizures or subclinical electrographic seizures were at increased risk of language delay (Rappaport et al. 1998; Clancy et al. 2003, 2005; Gaynor et al. 2007). However, it is not clear how non-convulsive or nonclinical seizures should be managed in this population (Naim et al. 2015). It may be important to recognize the prevalence and predictors of nonclinical seizure activity in this population and determine if this is a potentially modifiable risk factor for language delay. There has been discussion in the literature of monitoring and potential management strategies for non-convulsive seizures in post-operative cardiac surgery patients, but no randomized controlled trials have been published to date (Naim et al. 2015).

Near-infrared spectroscopy (NIRS) has recently been introduced to the clinical environment to measure cerebral oxygenation but these data are not available for the entire cohort. In addition, data related to clinical management decisions based on NIRS were not available. If NIRS leads to a consistent change in preoperative management with subsequent increase in cerebral oxygenation then there is the potential to improve long term neurodevelopmental outcome. In future studies, it will be important to prospectively collect these data as well as the specific clinical care NIRS protocols and management decisions based on NIRS.

The strengths of this study are the high follow up rate, with 93 % of children who underwent ASO included. The prospectively collected predictive variables using standardized study protocols and the experienced research teams that have worked together since 1996 are also considerable strengths. Limitations of this study are that data on some potential confounding factors are lacking or inconsistently collected including indication for BAS, initial and preoperative saturations, indications for delays in surgery and perioperative cerebral oxygenation through NIRS measurements. In addition, assessment by a speech-language pathologist was not possible at all sites and the children identified with language delay may represent an underestimate of the proportion of children with speech and language difficulties. In future studies, measures other than the Bayley-III Language Composite, administered by speech language pathologists, should be considered to better understand the nature of the language delay. While there were no comparison children, standardized measures with population-normative data were administered by experienced clinicians.

## Conclusions

Following ASO for TGA, children have cognitive and motor skills similar to population norms. Overall outcomes are good, but there continue to be important differences in language development with a risk of language delay at 2 years of age. This may be a marker for more concerning developmental and mental health issues as the child ages. As such, children who undergo neonatal cardiac surgery should receive regular developmental surveillance. Supporting families and children post-operatively with early intervention may help improve language outcomes. There were no differences in pre-operative or demographic variables, nor were there differences in cognitive, language or motor outcomes, based on the presence of a VSD. The rate of BAS was high but this was not a predictor of outcome. While perioperative factors account for some differences seen in language outcomes patient characteristics, particularly maternal education, are the strongest predictors of delay. There may be opportunities for pre and/or post-operative neuro-imaging and perioperative NIRS and EEG monitoring to better understand the timing of brain injury and identify potential therapeutic targets and modifiable risk factors. Additionally, prospective studies are required to understand the optimal timing for surgery.

## Abbreviations

ASO: arterial switch operation
BAS: balloon atrial septostomy
Bayley-III: Bayley Scales of Infant & Toddler Development—3rd Edition
BSID-II: Bayley Scales of Infant Development—2nd Edition
CPB: cardiopulmonary bypass
DHCA: deep hypothermic circulatory arrest
NEC: necrotizing enterocolitis
PICU: pediatric intensive care unit
TGA: transposition of the great arteries
VSD: ventricular septal defect
